# Risk factors for latent tuberculosis infection in RA patients treated with anti-tumor necrosis factor

**DOI:** 10.1186/ar3641

**Published:** 2012-02-09

**Authors:** Shiang-Fen Huang, Wei-Juin Su, Sheng-Yuan Ruan, Chong-Jen Yu, Song-Chou Hsieh, Yu-Chih Liu, Yeong-Jian Jan Wu, Hsiao-Yi Lin

**Affiliations:** 1 Department of Chest Medicine, Taipei Veterans General Hospital, School of Medicine, National Yang-Ming University, Taipei, Taiwan; 2 Division of Pulmonary and Critical Care Medicine, Department of Internal Medicine, National Taiwan University Hospital, Taipei, Taiwan; 3 Division of Allergy, Immunology and Rheumatology, Department of Internal Medicine, National Taiwan University Hospital, Taipei, Taiwan; 4 Department of Medicine, Division of Pulmonary Medicine, Chang Gung Memorial Hospital, Keelung, Taiwan; 5 Department of Medicine, Division of Allergy, Immunology and Rheumatology, Chang Gung Memorial Hospital, Keelung, Taiwan; 6 Division of Allergy, Immunology and Rheumatology, Department of Medicine, Taipei Veterans General Hospital, Taipei, Taiwan

## Background

To estimate the prevalence of latent tuberculosis (TB) infection according to the interferon-gamma release assay (IGRA, QuantiFERON^®^-TB Gold In-Tube, QFT) in patients with rheumatoid arthritis (RA), and assess the risk factors for incidence of active TB after TNF *alpha *blocking agents treatment.

## Methods

A multicenter, prospective, and observational study was started in April, 2011 for patients with RA in Taiwan University Hospital, Taipei Veterans General Hospital, and Chang Gung Memorial Hospital in Keelung. Patients who take anti-TNFα regiments or not (defined as naïve or never take agent) were both enrolled in the study. The clinical history, DAS-28 score, chest film finding, sputum survey for active TB, and QFT screening results were collected.

## Results

A total of 147 patients were enrolled in the study, in which five of them (3.4%) had history of anti-TB treatment and none had active TB at the beginning of the investigation. There were 75 patients undergoing anti-TNFα treatment before the study (42 patients (56%) took etanercepts and the other 33 (46%) ones took adalimumabs) and 72 patients had not (Table 1).

Based on QFT test, the frequency of latent TB infection (LTBI) were 12.5% (9/72) for naïve patients, and 10.7% (8/75) for biologics users (p > 0.05). Risk analysis showed no difference between different QFT results in study patients (Table 2).

The interval between starting etanercepts or adalimumabs treatment and screening for QFT test were 22.5 and 14.4 months (p > 0.05), respectively. Subgroup analysis showed possible risk factors for LTBI in patients who had history of adalimumabs or etanercept treatment were the history of anti-TB treatment and negative for BCG scar, respectively (p < 0.05). Other factors including DAS-28 score, presence of rheumatoid factor, white cell count, and previous immunosuppressant dosage (ie, prednisolone and methotrexate) were not related to the LTBI status (Table 3).

More patients had indeterminate QFT result after entracept treatment but negative QFT result after adalimumab therapy (*p*<0.05). In current study, none of patients with positive or indeterminate QFT result received preventive INH treatment and none of them had evidence of non-tuberculosis *mycobacterium *infection.

## Conclusion

The overall frequency of LTBI in patients with RA was 11.6% in this study. Although history of anti-TB treatment and negative BCG scar were risk factors for LTBI, other factors still need to be considered due to limited sample size in current study. Further regular follow up should be done.

